# Development and Validation of Deep Learning Model for Intermediate-Stage Hepatocellular Carcinoma Survival with Transarterial Chemoembolization (MC-hccAI 002): a Retrospective, Multicenter, Cohort Study

**DOI:** 10.7150/jca.91501

**Published:** 2024-02-17

**Authors:** Yaying Chen, Yanhong Shi, Ruiqi Wang, Xuewen Wang, Qin Lin, Yan Huang, Erqian Shao, Yan Pan, Shanshan Huang, Linbin Lu, Xiong Chen

**Affiliations:** 1 Department of Oncology, Mengchao Hepatobiliary Hospital of Fujian Medical University, Fuzhou, Fujian, China.; 2 Department of Gastroenterology, Xiamen Humanity Hospital, Xiamen, China.; 3 Department of Oncology, The 900th Hospital of the People's Liberation Army Joint Service Support Force, Fuzong Clinical Medical College of Fujian Medical University, Fuzhou, Fujian, China.; 4 Department of Oncology, Quanzhou First Hospital Affiliated to Fujian Medical University, Quanzhou, Fujian, China.; 5 Department of Oncology, The Third Affiliated Hospital of Sun Yat-sen University Yuedong Hospital, Meizhou, Guangdong, China.; 6 Department of Oncology, Fujian Provincial Hospital, Fuzhou, Fujian, China.; 7 Department of Oncology, The First Affiliated Hospital of Nanchang University, Nanchang, Jiangxi, China.

**Keywords:** hepatocellular carcinoma, transarterial chemoembolization, deep learning model, machine learning, deepHAP IV model

## Abstract

**Background**: There are few effective prediction models for intermediate-stage hepatocellular carcinoma (IM-HCC) patients treated with transarterial chemoembolization (TACE) to predict overall survival (OS) is available. The learning survival neural network (DeepSurv) was developed to showed a better performance than cox proportional hazards model in prediction of OS. This study aimed to develop a deep learning-based prediction model to predict individual OS.

**Methods**: This multicenter, retrospective, cohort study examined data from the electronic medical record system of four hospitals in China between January 1, 2007, to December 31, 2016. Patients were divided into a training set(n=1075) and a test set(n=269) at a ratio of 8:2 to develop a deep learning-based algorithm (deepHAP IV). The deepHAP IV model was externally validated on an independent cohort(n=414) from the other three centers. The concordance index, the area under the receiver operator characteristic curves, and the calibration curve were used to assess the performance of the models.

**Results**: The deepHAP IV model had a c-index of 0.74, whereas AUROC for predicting survival outcomes of 1-, 3-, and 5-year reached 0.80, 0.76, and 0.74 in the training set. Calibration graphs showed good consistency between the actual and predicted OS in the training set and the validation cohort. Compared to the other five Cox proportional-hazards models, the model this study conducted had a better performance. Patients were finally classified into three groups by X-tile plots with predicted 3-year OS rate (low: ≤ 0.11; middle: > 0.11 and ≤ 0.35; high: >0.35).

**Conclusion**: The deepHAP IV model can effectively predict the OS of patients with IM-HCC, showing a better performance than previous Cox proportional hazards models.

## Background

Intermediate-stage hepatocellular carcinoma (IM-HCC) has a wide heterogeneity in tumor burden, and the large gaps in residual liver function (Child-Pugh score 5 to 9), contributing to its highly variable lifetime. The combination of chemotherapy, programmed death 1/programmed death ligand 1 (PD-1/PD-L1) inhibitors, targeted drugs and t ransarterial chemoembolization (TACE) has become prominent in unresectable-HCC research. TACE is recommended as the frontline treatment for IM-HCC^1^, but only a subgroup of patients can benefit from this therapy. Therefore, it is necessary to establish a model to make an individualized prediction of the survival prognosis of this group of patients and identify the survival differences between patients. Several risk prediction models for HCC have been developed, usually based on Cox regression analysis, to identify individual clinical outcome, including up-to-seven criteria^2^, six-and-twelve criteria^3^, HAP score^4^, mHAP-Ⅱ score^5^, mHAP-Ⅲ^6^, ALBI grade^7^, BCLC-B HCC sub-classification^8^ and so on. However, most of these models are linear prediction models, and the relationship between each variable of real-world data is usually nonlinear^9^, ^10^. Some variables may be removed when constructing the above model, which causes partial distortion of the model and affects the prognosis.

Artificial intelligence (AI) can synthesize and analyze multimodal data with superhuman precision and reliability. In recent years, the use of AI in multiple medical fields, including liver disease, has rapidly increased^11^, ^12^. Katzman et al. developed a novel deep learning method for survival analysis that first uses a deep learning network to integrate Cox proportional hazards, which is referred to as the learning survival neural network (DeepSurv)^13^. Compared with the Cox proportional hazards model, DeepSurv model has demonstrated its superior performance in predicting prognosis and providing personalized treatment recommendations on multiple solid tumors^14^-^18^. However, few DeepSurv models are developed to identify the prognosis of this group of patients with strong heterogeneity in IM-HCC. In this study, we update a deepHAP Ⅳ model based on DeepSurv using five variables from mHAP-Ⅲ model to predict individual overall survival (OS) in IM-HCC.

## Methods

### Data sources and patient selection

Between January 1, 2007 to December 31, 2016, the consecutive unsectable HCC patients (BCLC stage B) treated with TACE as first-line therapy were retrospectively collected from the electronic medical record system of four hospitals in Guangzhou, China. Details of this multicenter, retrospective, cohort study were previously published in details^19^-^21^. The SYSUCC cohort included the HCC patients diagnosed at Sun Yat-sen University Cancer Center (SYSUCC) from January 2007 to December 2015. And the multicenter cohort included the patients from three another hospitals between January 2010 and December 2016.

Clinical cases were included if patients satisfied the following criteria: clinically diagnosis of BCLC stage B HCC; complete data of the following at initial diagnosis (computerized tomography (CT) or magnetic resonance imaging (MRI) of the abdominal region, radiography or CT of the chest, routine bloodwork test, biochemical routine test, serum alpha-fetoprotein (AFP) level, and coagulation indices); no history of other malignancies. Patients were excluded for refusing to receive treatment (n=37), or treating with surgical resection ate 1st line (n=225).

The research was carried out under the guidance of the Declaration of Helsinki. The Clinical Research Department approved the study protocol (2017-FXY-129) of SYSUCC. The informed consent was waived for this study as a secondary analysis study, and patients in the study were anonymized.

### Definitions of variables and outcomes

Only baseline data including age, gender, AFP, albumin (ALB), total bilirubin (TBLT), Child-Pugh class, major tumor size, location of Lesions, intrahepatic lesions number, and hepatitis B virus (HBV) infection, were collected in the analysis. The distribution of AFP and TBLT were skewed towards the left and transformed to the Log10 scale (Log AFP and Log TBLT) for analysis. According to the mHAP II score^5^ and mHAP-III model^6^, continuous variables were divided into categorical variables, including AFP (≤400, >400), major tumor size (≤ 7, >7), No. of intrahepatic lesions (2, 3, >3). The interesting endpoint was OS, which the time from the first diagnosis of HCC to death or last follow-up.

### Deep learning model design and statistical analysis

In this study, some continuous variables were transformed into categorical variables, expressed in terms of number and proportion. Continuous variables were included in the study either in logarithmic form or by calculating the standard deviation of the mean. Differences are compared using the chi-square test (or Fisher's exact test) for categorical measures and Kruskal-Wallis test for continuous measures. Univariate COX regression analysis was performed to explore the relationship between baseline characteristics and OS. To be consistent with mHAP-III model, Log AFP, ALB, Log TBLT, major tumor size, and intrahepatic lesion number were taken into DeepSurv neural network model^13^, namely the deepHAP IV model.

**Figure 1:**
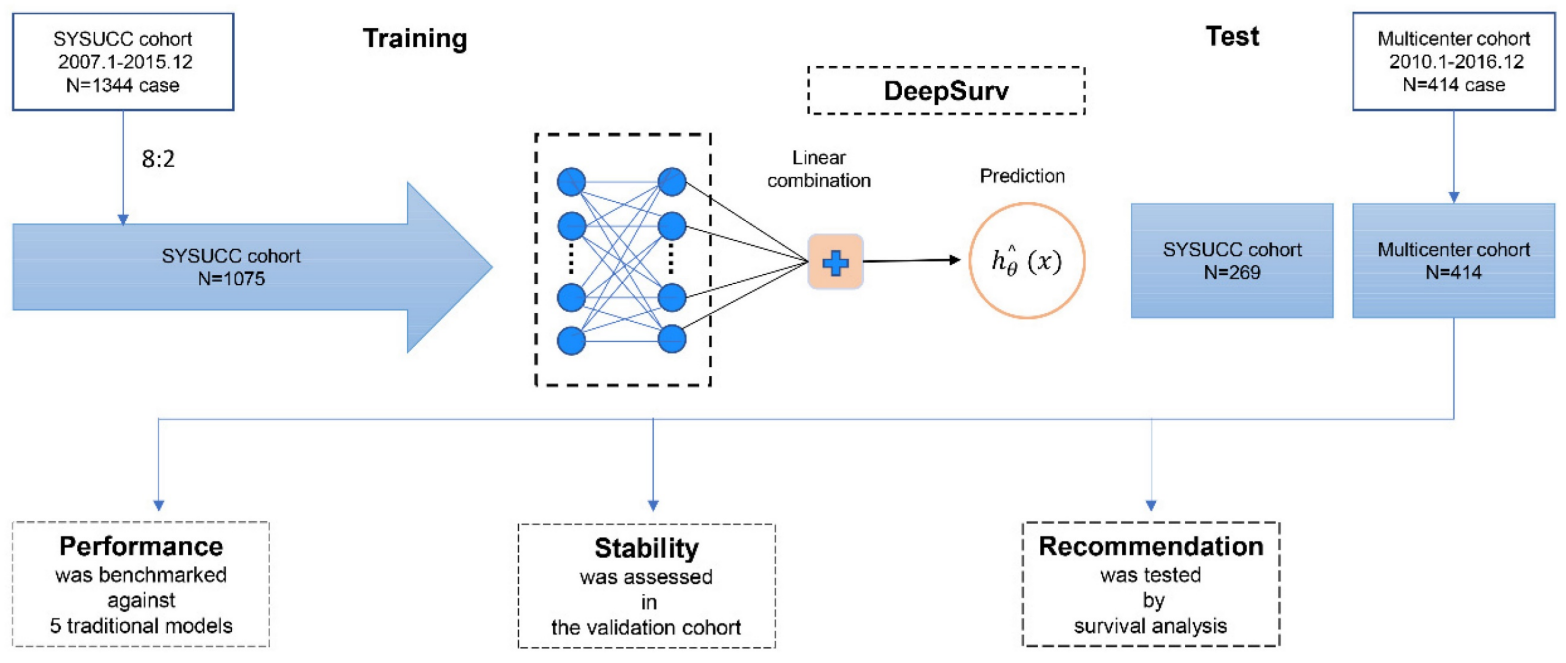
Diagram of the Study Procedure.

A deep learning algorithm with a 2-layer neural network was used to establish a prediction model which can be more individualized to predict OS with IM-HCC. Convolutional neural networks (CNN) are a class of feedforward neural networks with deep structure and convolutional computation. In this study, we conducted the deep learning model by CNN. The deepHAP IV model contained a single output node to calculate patients' survival risks hθ(xi) using the negative log-partial likelihood function (Figure 1).

Model discrimination was evaluated using C-index and time-area under curve (t-AUC) curves. Calibration plots assess the degree of agreement between the model's predicted and observed probability. We aimed to assess the clinical practicability of our model by comparing its performance with existing metrics, including HAP score, mHAP II score, Up-to-seven, Four-and-seven, and Six-and-twelve.

X-tile software was used to distinguish patients at low and intermediate survival risk from those at high risk. Kaplan-Meier curves and log-rank tests were used to compare differences between groups. A 2-sided P<0.05 was statistically significant. Statistical analyses were mainly performed using R language software (version 4.2.2; Vienna, Austria; Fig. www.r-project.org) and X-tile software (Yale University School of Medicine, New Haven, CT, USA).

## Results

### Patient Characteristics

A total of 8848 cases and 1695 cases of HCC patients were collected in SYSUCC and multicenter. We screened the inclusion and exclusion criteria and finally collected 1344 cases of clinical data from the SYSUCC cohort. Among them, 1075 patients were trained to establish the deepHAP IV model, and 269 patients were used as the internal test set at 8:2 ratio. Besides, 414 from the multicenter cohort were finally enrolled in the external cohort (Figure S1). The baseline characteristic was shown in Table 1. Most patients were Child-Pugh class A (1502/1758, 85.4%), and the median tumor size was 7.2cm. Most of the patients (1662/1758, 94.5%) had a history of HBV infection.

### Development of deepHAP IV model and validation

Table 2 showed the univariate COX regression analysis results for potential risk factors. Considering the HAP-Ⅲ model, we finally selected five factors, including log AFP, ALB, Log TBLT, major tumor size and intrahepatic lesion number, to develop a deepHAP IV model. The training set reached a C-index of 0.74 (95%CI: 0.71, 0.77), with a 1-, 3-, and 5-year area under the receiver operating characteristic (AUROC) is 0.80, 0.76, and 0.74, respectively. We have listed 5 samples of predicted survival rates for IM-HCC treated with TACE. For example, a 66-year-old patient with a Child-Pugh score of 6, his AFP at baseline is 792.6 ng/ml, ALB is 34.7 g/L, TBLT is 13.4μmol/L, largest tumor size is 4cm, tumor number is 2, by using our model, he got a survival rate at 1-, 3-, 5-year is 0.819, 0.519 and 0.386. While his actual OS is 12.8 months, which indicates our model deed has a good prediction power (Table 3).

**Figure 2:**
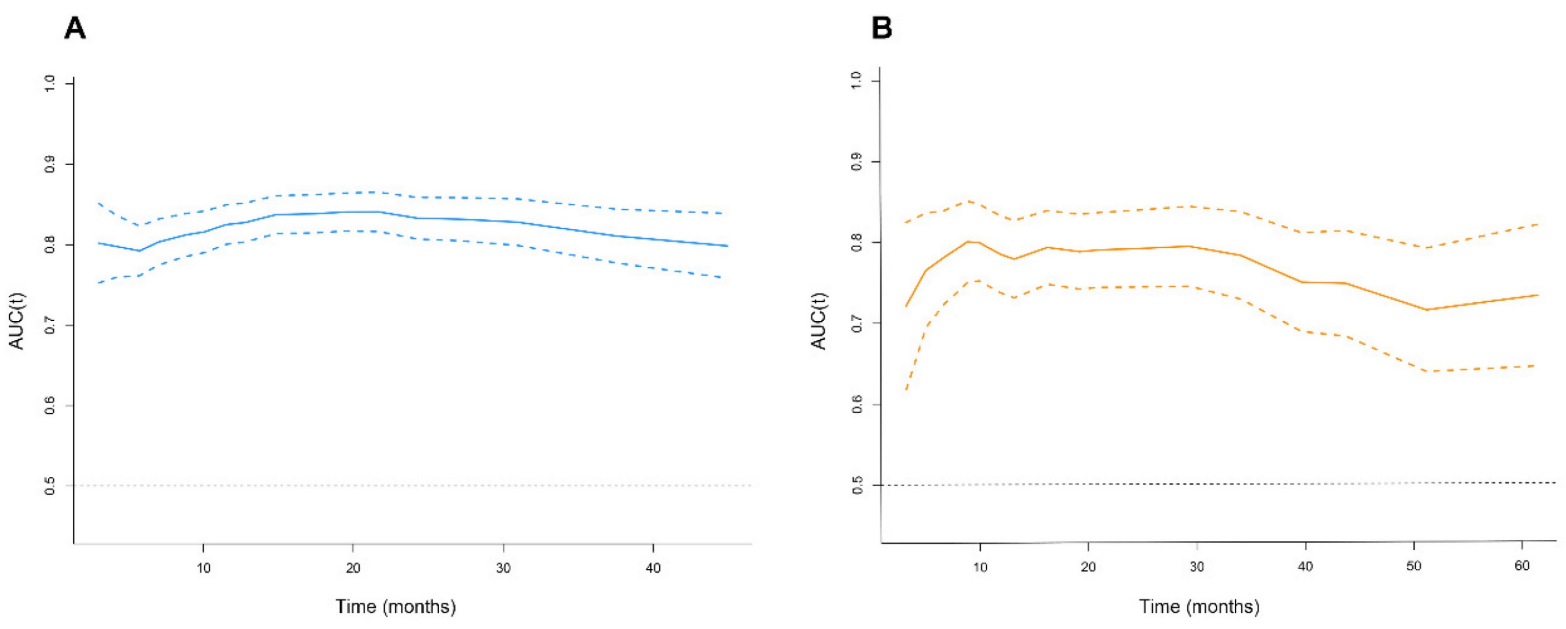
The time-dependent area under the curve in the SYSUCC Cohort (A) and Multicenter Cohort (B).

The test and external validation cohorts were set independently for internal and external validation. The C-index of test set was 0.69 (0.63, 0.76), and the 1-, 3-, 5-year AUROC is 0.74, 0.70, 0.69. The C-index of validation cohort was 0.70 (0.65, 0.75), and the 1-, 3-, 5-year AUROC is 0.77, 0.73, 0.70. Besides, a further time-dependence receiver operating characteristic (ROC) showed a stable performance in prediction power in 40 months both in the training set and validation cohort. When the time stretch to 40 months, we can still see continuous stability in the SYSUCC cohort, but the stability in the multicenter cohort is less satisfactory (Figure 2).

A comparison between our model with five others 2-5, 22, including the HAP and mHAP II scores, as well as the up-to-seven, the four-and-seven, the six-and-twelve score, indicated that ours had the highest C-index and 1-, 3-, 5-years AUROC (Table 4).

**Figure 3:**
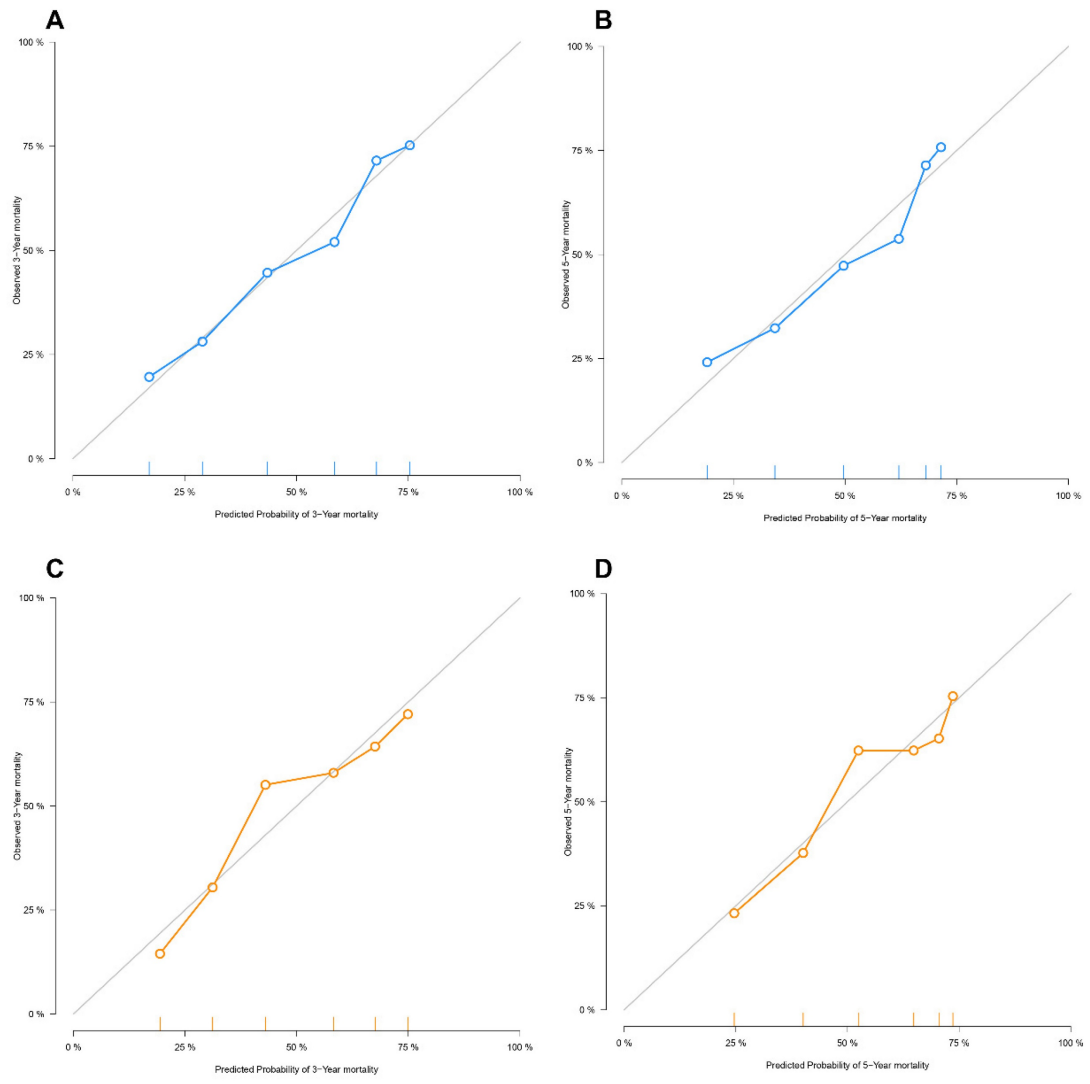
Calibration Plots for Overall Survival for the deepHAP IV Model in the SYSUCC Cohort (A, B) and Multicenter Cohort (C, D).

A calibration curve was performed in the training set and validation cohort of 3-year and 5-year OS in IM-HCC patients. Calibration curves showed good consistency between the prediction model's actual and predicted survival rates (Figure 3).

**Figure 4:**
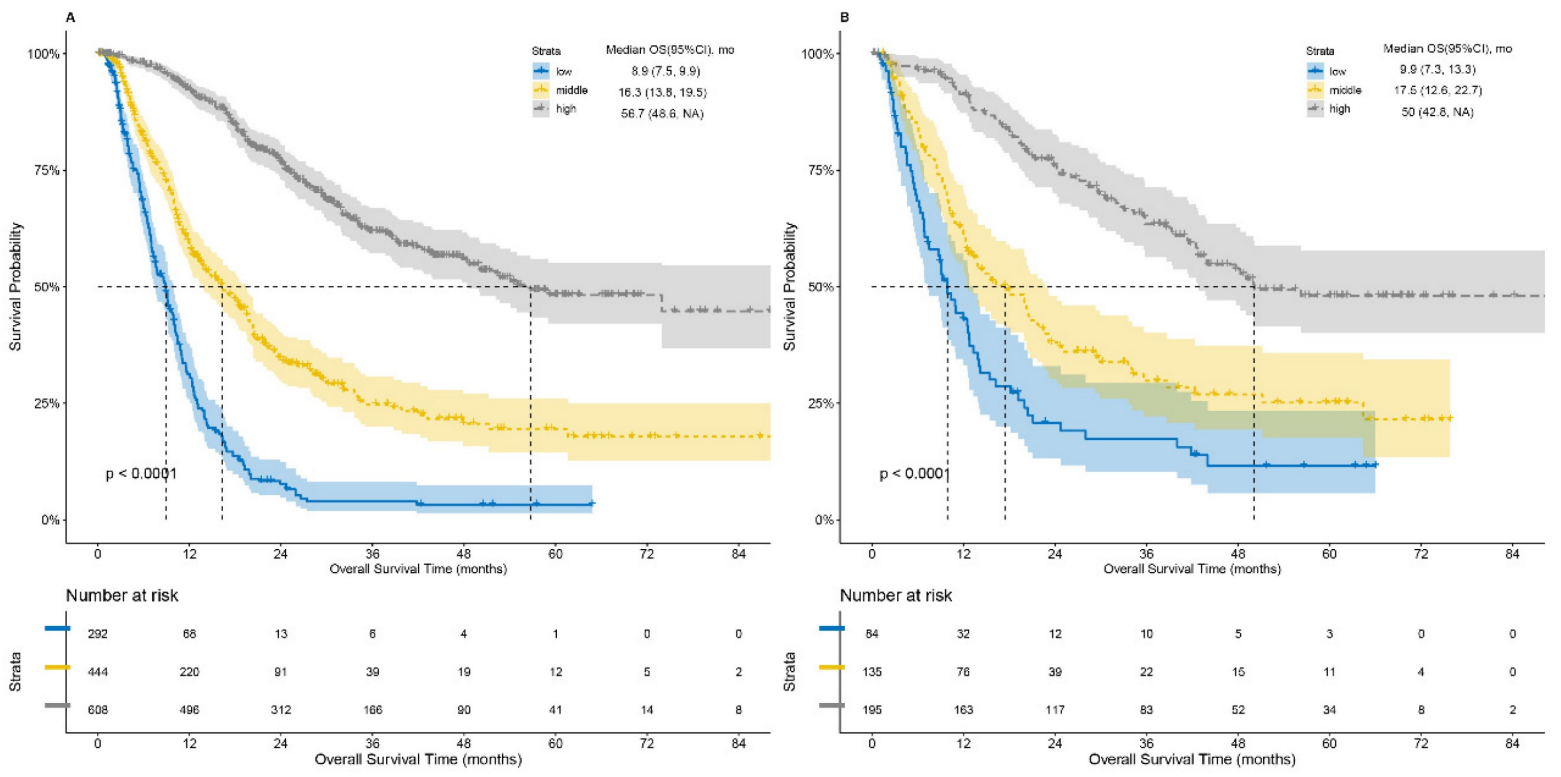
Kaplan-Meier curves of overall survival stratified by predicted 3-year survival rate. low: ≤ 0.11; middle: > 0.11 and ≤ 0.35; high: >0.35.

Individual patient 3-year survival rates were calculated according to the deepHAP IV model, and patients were divided into a low survival rate group (3-year survival rate ≤0.11), a moderate survival rate group (0.11 <3-year survival rate ≤ 0.35), and a high survival rate group (3-year survival rate >0.35) using X-tile. Significant differences in OS in both the training set and validation cohort were observed among the three groups (p <0.0001) (Figure 4). The OS in the training set was 8.9, 16.3, and 56.7 months in the low, middle, and high groups, respectively.

## Discussion

In this study, we constructed a model based on a deep learning algorithm that can individualize and predict the survival prognosis of patients with IM-HCC after TACE. Five parameters, log AFP, ALB, Log TBLT, major tumor size, and intrahepatic lesion number, were assessed and identified as predictors and used in model construction. By comparing several linear prediction models, it is proved that the prediction performance of the deepHAP IV model is better than that of traditional linear prediction models. This model effectively assesses the prognosis after TACE in patients with BCLC stage B HCC. Besides, populations were divided into three groups by predicted 3-year survival rate (low: ≤ 0.11; middle: > 0.11 and ≤ 0.35; high: >0.35).

Several common liner models, such as HAP score^4^, mHAP-Ⅱ score^5^, up-to-seven criteria^2^, six-and-twelve criteria^3^ and four-and-seven^22^, are often used to compare model performance. L. Kadalayil et al. firstly developed a simple liner model to predict OS of HCC patients with TACE, named HAP, and proved to be better performance against the other prognostic model^4^. We performed it with our data and obtained a less satisfactory performance with a C-index of 0.63, and 1-, 3-, 5-years AUROC of 0.67, 0.63, 0.58. Lin H also tried this model with itself data, showed even poor performance than ours with a C-index of 0.54, and 1-, 3-years AUROC of 0.60, 0.58^23^. In Park's et al study, by adding the "tumor number" variable to HAP model, mHAP-II was constructed and got a better prediction performance. But when we use our queues to validate both models, the expressiveness is similar^5^. Up-to-seven criteria is an expanded criteria beyond to Milan criteria which can better predict the OS after liver transplantation in patients with HCC^2^. In our research, this model acquired a 1-, 3-, 5-years AUROC at 0.62, 0.64 and 0.63, C-index at 0.60. Lin H also tested with their data and got a AUROC of 0.62 in both 1-year and 3-years, a C-index of 0.59^23^. The same with models mentioned above, the six-to-twelve score and four-and-seven also showed poor performance in our and Lin H' research. In a word, we compared several liner models, and found prediction model contrasted by ML method can perform better. Liner models may have some limitation such as over-fitting or non-liner relation between variables^10^, ^24^.

Many studies have developed prognostic, predictive models for HCC using the ML method with the development of ML algorithms. Lin H has explored a machine learning-based model to predict the survival prognosis of patients with IM-HCC after TACE. Five variables were included in the model: the size of the tumor, BCLC B sub-classification, AFP, ALB, and the number of lesions. The established model had a C-index of 0.69, whereas AUROC for predicting survival outcomes of the first three years reached 0.72, 0.71, and 0.73^23^. The performance of their model is less satisfactory. Hence, we developed the deepHAP IV model in the study with a c-index of 0.74, whereas AUROC for predicting survival outcomes of 1-, 3- and 5 years reached 0.80, 0.76, and 0.74 in the training set. The calibration curve and standard line coincide well. And we further divided it to high 3-year survival rate, middle rate and low rate group. Deep learning has already been applied to multiple tumor species. Deep learning networks can learn the highly intricate and linear/nonlinear associations between prognostic clinical characteristics and an individual's risk of death from HCC-specific survival^25^.

This study had several limitations. As a secondary study, the raw data's limitations, such as selective bias which is one of the intrinsic limitations of retrospective data, cannot be avoided. The data were derived from a Chinese population, and it remains to be verified whether the model applies to other ethnic populations. Second, Clinically, there may be some other indicators that can also affect prognosis, but these indicators are missing from the original data, so they are not included in this study model. More clinical parameters, genetics, and imaging features need to be informative in the modeling in the future. As methods of TACE and times of TACE can influence the OS of IM-HCC, it's important to including information about these. We hope to conduct more prospective clinical studies on IM-HCC in the future to confirm our conclusions and hypotheses. Third, the current deep learning methods are still not readily available for clinical practice. Finally, it is difficult to understand how the deep learning network makes its decisions, for the networks function much like black boxes.

In conclusion, we established a deep learning-based model, which can effectively predict the OS of patients with IM-HCC, showing a better performance than previous Cox proportional hazards models.

## Supplementary Material

Supplementary figure.

## Figures and Tables

**Table 1:** Baseline characteristics of training and validation cohort.

	Training set	Test set	Validation cohort	P-value
N	1075	269	414	
Age(yr)	53.8 ± 12.1	53.1 ± 12.3	51.6 ± 12.0	0.006
Gender				<0.001
Male	646 (60.1%)	150 (55.8%)	348 (84.1%)	
Female	429 (39.9%)	119 (44.2%)	66 (15.9%)	
ALB (g/L)	38.6 ± 5.7	38.7 ± 5.5	38.9 ± 5.7	0.725
Log TBLT (μmol/L)	1.2 ± 0.3	1.3 ± 0.3	1.3 ± 0.3	0.026
AFP (ng/ml)				0.114
≤400	583 (54.2%)	130 (48.3%)	233 (56.3%)	
>400	492 (45.8%)	139 (51.7%)	181 (43.7%)	
Log AFP	2.5 ± 1.4	2.6 ± 1.4	2.3 ± 1.4	0.007
Child-Pugh class				0.337
A	928 (86.3%)	229 (85.1%)	345 (83.3%)	
B	147 (13.7%)	40 (14.9%)	69 (16.7%)	
Major tumor size(cm)				0.125
≤7	594 (55.3%)	130 (48.3%)	224 (54.1%)	
>7	481 (44.7%)	139 (51.7%)	190 (45.9%)	
Mean±SD	7.2 ± 3.6	7.5 ± 3.9	7.1 ± 3.5	0.328
Location of Lesions				0.115
Unilobar	423 (39.3%)	104 (38.7%)	186 (44.9%)	
Bilobar	652 (60.7%)	165 (61.3%)	228 (55.1%)	
Intrahepatic lesions number				0.071
2	296 (27.5%)	73 (27.1%)	141 (34.1%)	
3	89 (8.3%)	17 (6.3%)	25 (6.0%)	
>3	690 (64.2%)	179 (66.5%)	248 (59.9%)	

Mean+SD/N(%). Differences are compared using the chi-square test (or Fisher's exact test) for categorical measures and Kruskal-Wallis test for continuous measures.

**Table 2:** Univariate Cox regression analysis of potential risk factors

	Statistics	HR (95% CI)	*P*-value
AGE			
≤55	941 (53.5%)	1	
>55	817 (46.5%)	1.07 (0.94, 1.22)	0.317
Gender			
male	1144 (65.1%)	1	
female	614 (34.9%)	0.82 (0.72, 0.95)	0.008
AFP (ng/ml)			
≤400	946 (53.8%)	1	
>400	812 (46.2%)	1.49 (1.31, 1.70)	<0.0001
Log AFP	2.5 ± 1.4	1.21 (1.15, 1.27)	<0.0001
ALB (g/L)	38.7 ± 5.7	0.98 (0.97, 0.99)	0.0004
Log TBLT (μmol/L)	1.3 ± 0.3	1.21 (0.97, 1.52)	0.097
Child-Pugh class			
A	1502 (85.4%)	1	
B	256 (14.6%)	1.44 (1.21, 1.71)	<0.0001
Diameter of main tumor(cm)			
≤ 7	948 (53.9%)	1	
>7	810 (46.1%)	2.40 (2.10, 2.74)	<0.0001
Mean±SD	7.2 ± 3.6	1.14 (1.12, 1.16)	<0.0001
Location of Lesions			
Unilobar	713 (40.6%)	1	
Bilobar	1045 (59.4%)	1.57 (1.37, 1.79)	<0.0001
No. of intrahepatic lesions			
2	510 (29.0%)	1	
3	131 (7.5%)	1.26 (0.96, 1.67)	0.098
>3	1117 (63.5%)	1.76 (1.51, 2.06)	<0.0001
HBV			
No	96 (5.5%)	1	
Yes	1662 (94.5%)	1.01 (0.75, 1.36)	0.940

**Table 3:** Five samples of predicted survival rate for intermediate-stage HCC treated with TACE

Samples	AFP (ng/ml)	ALB (g/L)	TBLT (μmol/L)	Largest tumor size (cm)	Tumor number	Predicted 1-yr survival rate	Predicted 3-yr survival rate	Predicted 5-yr survival rate
A 66-year-old man, Child-Pugh, scored 6. OS 12.8 months and dead.	792.6	34.7	13.4	4	2	0.819	0.519	0.386
A 75-year-old man, Child-Pugh score 5. OS is 6.7 months and dead.	32.2	38.6	13.4	7.2	3	0.687	0.292	0.167
A 41-year-old woman, Child-Pugh, scored 6. OS is 5.5 months and dead.	23.6	34.6	19.6	7.2	>3	0.641	0.232	0.12
A 44-year-old man, Child-Pugh score 5. OS 3 months and dead.	53553	43.7	11.7	10	>3	0.615	0.202	0.098
A 33-year-old man, Child-Pugh score 5. OS 4.3 months and dead.	121000	48.1	11	120	2	0.508	0.108	0.039

**Table 4:** The comparison of the deepHAP IV model versus other models for intermediate-stage HCC treated with TACE.

Overall Survival	Model	1-yr AUROC	3-yr AUROC	5-yr AUROC	Harrell's C statistic (95% CI)
Training set	HAP	0.67	0.63	0.58	0.63(0.58, 0.67)
	mHAP II	0.67	0.63	0.58	0.63(0.58, 0.67)
	Up-to-seven	0.62	0.64	0.63	0.60(0.57, 0.63)
	Four-and-seven	0.62	0.64	0.63	0.60(0.57, 0.63)
	Six-and-twelve	0.66	0.62	0.57	0.63(0.59, 0.67)
	**deepHAP IV**	**0.80**	**0.76**	**0.74**	**0.74(0.71, 0.77)**
Test set	HAP	0.67	0.63	0.62	0.64(0.54, 0.73)
	mHAP II	0.67	0.63	0.62	0.64(0.54, 0.73)
	Up-to-seven	0.62	0.59	0.58	0.60(0.54, 0.65)
	Four-and-seven	0.62	0.59	0.58	0.60(0.54, 0.65)
	Six-and-twelve	0.64	0.61	0.60	0.63(0.55, 0.71)
	**deepHAP IV**	**0.74**	**0.70**	**0.69**	**0.69(0.63, 0.76)**
Validation cohort	HAP	0.67	0.63	0.58	0.62(0.55, 0.69)
	mHAP II	0.67	0.63	0.58	0.62(0.55, 0.69)
	Up-to-seven	0.63	0.61	0.58	0.59(0.55, 0.64)
	Four-and-seven	0.63	0.61	0.58	0.59(0.55, 0.64)
	Six-and-twelve	0.70	0.63	0.62	0.63(0.57, 0.64)
	**deepHAP IV**	**0.77**	**0.73**	**0.70**	**0.70(0.65, 0.75)**

## Abbreviations

IM-HCC: intermediate-stage hepatocellular carcinoma
HCC: hepatocellular carcinoma
TACE: transarterial chemoembolization
OS: overall survival
PD-1/PD-L1: programmed death 1/programmed death ligand 1
BCLC: Barcelona Clinic Liver Cancer
AI: artificial intelligence
SYSUCC: Sun Yat-sen University Cancer Center
CT: computerized tomography
MRI: magnetic resonance imaging
AFP: alpha-fetoprotein
ALB: albumin
TBLT: total bilirubin
HBV: hepatitis B virus
CNN: convolutional neural networks
t-AUC: time-area under curve
AUROC: area under the receiver operating characteristic
ROC: receiver operating characteristic
