# Supplementary figures and images for: Development and Validation of Deep Learning Model for Intermediate-Stage Hepatocellular Carcinoma Survival with Transarterial Chemoembolization (MC-hccAI 002): a Retrospective, Multicenter, Cohort Study
Source: J Cancer. 2024 Feb 17;15(7):2066–73. doi: 10.7150/jca.91501 (PMC10905396; doi:10.7150/jca.91501)

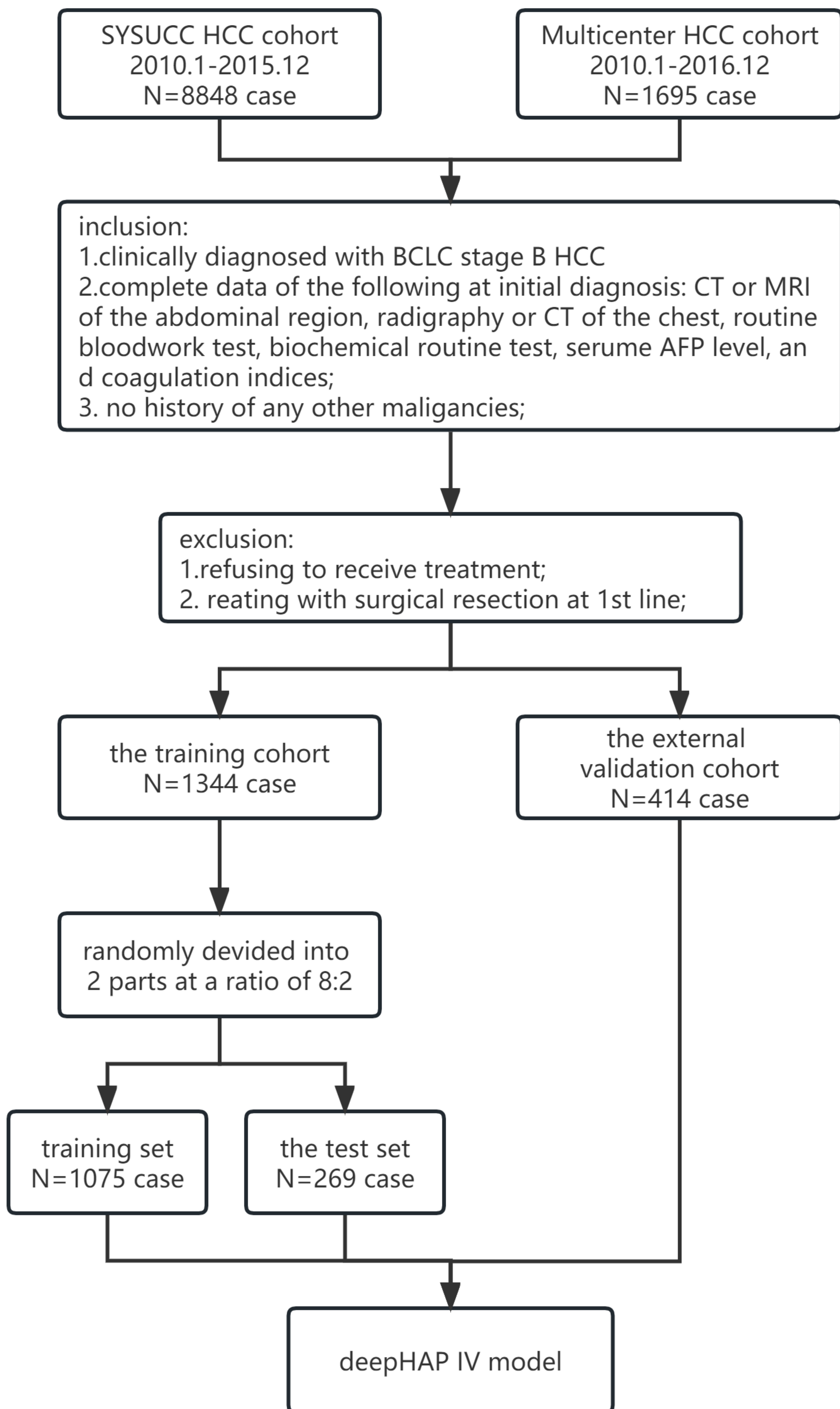

Supplement: Supplementary file 1 — Supplementary figure. [file jcav15p2066s1.pdf]
